# Geographical and Cultivar Features Differentiate Grape Microbiota in Northern Italy and Spain Vineyards

**DOI:** 10.3389/fmicb.2018.00946

**Published:** 2018-05-15

**Authors:** Valerio Mezzasalma, Anna Sandionigi, Lorenzo Guzzetti, Andrea Galimberti, Maria S. Grando, Javier Tardaguila, Massimo Labra

**Affiliations:** ^1^Zooplantlab, Department of Biotechnology and Biosciences, University of Milano-Bicocca, Milan, Italy; ^2^BEST4FOOD, University of Milano-Bicocca, Milan, Italy; ^3^Research and Innovation Centre, Fondazione Edmund Mach, San Michele all’Adige, Italy; ^4^Center Agriculture Food Environment (C3A), University of Trento, San Michele all’Adige, Italy; ^5^Instituto de Ciencias de la Vid y del Vino, University of La Rioja, CSIC, Rioja Regional Government, Logroño, Spain

**Keywords:** fruit microbiome, high throughput sequencing, soil microbiome, *Vitis vinifera*, wine

## Abstract

Recent studies have highlighted the role of the grapevine microbiome in addressing a wide panel of features, ranging from the signature of field origin to wine quality. Although the influence of cultivar and vineyard environmental conditions in shaping the grape microbiome have already been ascertained, several aspects related to this topic, deserve to be further investigated. In this study, we selected three international diffused grapevine cultivars (Cabernet Sauvignon, Syrah, and Sauvignon Blanc) at three germplasm collections characterized by different climatic conditions [Northern Italy (NI), Italian Alps (AI), and Northern Spain (NS)]. The soil and grape microbiome was characterized by 16s rRNA High Throughput Sequencing (HTS), and the obtained results showed that all grape samples shared some bacterial taxa, regardless of sampling locality (e.g., *Bacillus*, *Methylobacterium*, *Sphingomonas*, and other genera belonging to Alphaproteobacteria, Gammaproteobacteria, and Actinobacteria). However, some Operational Taxonomic Units (OTUs) could act as geographical signatures and in some cases as cultivar fingerprint. Concerning the origin of the grape microbiome, our study confirms that vineyard soil represents a primary reservoir for grape associated bacteria with almost 60% of genera shared between the soil and grape. At each locality, grapevine cultivars shared a core of bacterial genera belonging to the vineyard soil, as well as from other local biodiversity elements such as arthropods inhabiting or foraging in the vineyard. Finally, a machine learning analysis showed that it was possible to predict the geographical origin and cultivar of grape starting from its microbiome composition with a high accuracy (9 cases out of 12 tested samples). Overall, these findings open new perspectives for the development of more comprehensive and integrated research activities to test which environmental variables have an effective role in shaping the microbiome composition and dynamics of cultivated species over time and space.

## Introduction

In the last 10 years, due to the advances in metagenomics, it has become clearer and clearer that plants host a wide array of bacteria and yeasts (Melcher et al., 2014), most of which are not cultivable and therefore are almost unknown at the taxonomic and metabolic levels. Such microorganisms interact with the plant organs and are able to influence plant nutrition, development, productivity, and stress responses (White et al., 2014; Bacon and White, 2016).

Soil acts as a microbial reservoir for plants, especially concerning underground plant microbiota (Barata et al., 2012; Bacon and White, 2016). Usually, microbial diversity is higher at the roots than at aboveground organs due to the mostly selective nutrient-poor conditions and high exposure to variable abiotic factors (i.e., temperature, humidity, and UV radiation intensity) of the leaf, flower, and fruit (Ottesen et al., 2013). The origin of the microbial community in aboveground plant organs is less studied than that of the roots and many issues, such as the relationships among the microbiome of different plant organs or the influence of environmental microbial reservoirs (e.g., insect vectors and nearby plants), are still open (Compant et al., 2011; Berg et al., 2014).

It is even clearer that environmental microorganisms are essential for ensuring ecosystem equilibrium and are able to influence the relationships between plants and abiotic (e.g., soil, water, and solar light) and biotic (e.g., other microbial organisms) elements. Understanding how microbial assemblages colonizing the whole plant can play a key role in ecosystem and agroecosystem management is a challenging issue of emerging concern (van der Heijden and Wagg, 2013). In the last few years, one of the main research goals was discovering the origin of microbial community that colonizes crops and its direct influence on plant productivity, stress tolerance, and resistance (Morgan et al., 2017). This information may provide biological targets for future biotechnological applications, as well as basal information to control field microbial diversity for enhancing production yield (Finkel et al., 2017).

In the case of the grapevine (*Vitis vinifera* L.), the role of plant microbiota is much more relevant because field microorganisms have a documented effect during wine fermentation (Grangeteau et al., 2017; Mezzasalma et al., 2017) and act as signature of grape origin (Bokulich et al., 2014; Burns et al., 2015). Moreover, Bokulich et al. (2016) demonstrated that the microbial activity, combined with the abiotic and biotic factors, contributes to characterize wine *terroir*.

The grapevine naturally hosts a rich community of microorganisms that interact with plant organs, including fruit, and they can be transferred to the winery where, ultimately, they may affect wine chemical composition and influence its quality, even at the regional scale (Knight et al., 2015; Bokulich et al., 2016; Belda et al., 2017; Morgan et al., 2017). Recently, DNA studies supported that the grape microbiome is related to vineyard location, climatic conditions, and other vineyard-related factors (Taylor et al., 2014; Bokulich et al., 2016). Other studies also showed that agronomical practices, such as biodynamic management are able to modify the microbiome of grape and must (Burns et al., 2016; Mezzasalma et al., 2017; Morrison-Whittle et al., 2017). Similarly, other authors suggested that the occurrence of specific bacteria in must and wine has an effect on wine characteristics and typicity (Belda et al., 2017; Liu et al., 2017).

One of the main questions regards how the influence of grapevine cultivar and plant organs models the grape microbiome. Martins et al. (2013) showed that some epiphytic bacteria were shared by aerial plant portions and the soil. This finding led them to propose that the physical proximity between soil and the plant might facilitate microbial migration through rain splash, winds, pollinators and other foragers, and parasites. Compant et al. (2011) showed that grapevine’s aboveground organs might also be colonized by bacteria from other plants species. However, the molecular and physical mechanisms involved in plant–microbial interactions are not completely clear. Moreover, any grapevine cultivar show peculiar secondary metabolites, and most of these are concentrated in the fruit. Some of these metabolites have antimicrobial properties (Chong et al., 2009; Katalinić et al., 2010) and could influence the composition of grape microbiome both quantitatively and qualitatively. Based on these assumptions, we hypothesize that each cultivar could have an active and specific role in the interaction with and selection of its microbial community.

In this work, we investigated the microbiome composition of three international grapevine cultivars [i.e., Cabernet Sauvignon (CS), Sauvignon Blanc (SB), and Syrah (SY)] growing under three different geographical and environmental conditions. We characterized the composition of the grape microbiome of each cultivar and evaluated the influence of vineyard soils and grape characteristics in shaping plant epiphytic bacteria.

## Materials and Methods

### Plant and Soil Sampling

Cabernet Sauvignon, Syrah, and Sauvignon Blanc were chosen as candidate cultivars to evaluate the role of plant features in selecting surface bacteria. These are commonly widespread cultivars that differ in many characteristics, including the traits of bunch and berry (**Table 1**).

**Table 1:** Morphological characteristics of bunch and grape of the three studied cultivars.

Cultivar	Bunch			Berry				
	Compactness	Size (length/width)	Shape	Size (length/width)	Thickness of skin	Bloom	Consistency	Color
Cabernet Sauvignon	Medium/dense	Short/medium	Conical	Short/medium	Medium	High	Soft	Blue – dark
Syrah	Medium/dense	Short/medium	Funnel shaped	Medium/medium	Medium	Medium/high	Medium-hard	Blue – dark
Sauvignon Blanc	Dense	Medium/medium	Conical	Medium/medium	Medium	Medium	Soft	Green–yellow

*Data obtained from the Organisation Internationale de la Vigne et du Vin (OIV, http://www.oiv.int) and the Italian Vitis Database (VitisDB, http://www.vitisdb.it). Ampelometric characteristics were also verified by field visual inspection.*

To estimate the role of environmental conditions on these cultivars and specifically on their berry microbiome, we collected grape samples at three recognized grapevine germplasm collections having similar pedological features (i.e., gravelly sandy soil, with good drainage and permeability to water and air). The first sampling site was the germplasm collection of E. Mach Foundation (latitude 46°18′37″N; longitude 11°13′4″E), at the foot of the Italian Alps (hereafter AI) having the following soil characteristics, 52% sand, 39% loam and 9% clay, pH = 8.0 and organic matter about 20 g/kg. The second site was the Lombardy Regional Collection (latitude 44°58′35″’N, longitude 9°5′61″E), in Northern Italy (hereafter NI) characterized by mild continental climatic conditions and having the following soil features: 50% sand, 38% loam, 12% clay, pH = 8.2, organic matter = 16 g/kg. The last site was the experimental collection of Government of La Rioja (latitude 42°28′N, longitude 2°27′W), located in Northern Spain (hereafter NS) and characterized by a continental climate. The soil characteristics of this locality are 48% sand, 36% loam, 16% clay, pH = 8.7 and organic matter = 10 g/kg.

Each grapevine collection had at least three rows for each cultivar. During the harvest of 2016, five healthy and mature grape bunches (20°Bx) for each cultivar were collected from five scattered plants among rows. One-degree Brix is defined as 1 g of sucrose in 100 g of solution and this scale system is used as a proxy for grape maturation and fermentation progress. Sampling was performed in collaboration with specialized technicians from research institutes. Overall, a total of 45 grape samples (5 bunches × 3 cultivars × 3 germplasm collections) were collected.

Grape bunches were placed in sterile plastic bags and transferred to the laboratory in a refrigerated container. Under aseptic conditions, berries were harvested, gently destemmed, separating stems from berries, and pooled together. Grape pools (100 g each) were immediately frozen and stored at -80°C until DNA isolation.

Given the homogenous pedological characteristics at each germplasm collection, we collected five soil samples randomly distributed among the rows of the three studied cultivars. Each sample consisted of three soil top-layer (i.e., 0–10 cm) cores scattered in a range of 1 m and pooled together. On the whole, a total of 15 samples (i.e., 5 soil samples × 3 germplasm collections) were analyzed. Samples were stored at -80° C until DNA isolation.

### DNA Extraction

Microbial biomass recovery from grape samples was obtained starting from twenty berries randomly selected from each cultivar of each sampling site. Berries were thawed and placed in 500 mL sterile Erlenmeyer flasks and washed with 100 mL of isotonic solution (0.9% w/v NaCl) for 3 h with agitation at 150 rpm. The obtained cell suspension was separated from the berries by centrifugation at 6,000 ×*g* for 15 min. Pellets were stored at -20° C until DNA isolation. Total genomic DNA was obtained from pellets using PowerSoil^TM^ DNA Isolation Kit (MO BIO Laboratories, Carlsbad, CA, United States) following the manufacturer’s instructions with modifications specific for wet soil samples.

The same commercial kit was adopted to extract soil DNA starting at 0.25 g of soil for each collected sample. Before library preparation, the obtained genomic DNA extracts were purified using Zymo Research DNA Clean and Concentrator-10 (Zymo Research, Irvine, CA, United States) to remove PCR inhibitors.

### Library Preparation and Sequencing

DNA libraries for each sample were prepared following Illumina guidelines (16S Metagenomic Sequencing Library Preparation, Part #15044223 Rev. B) with modifications. Bacterial V3 and V4 regions of the 16S rRNA gene were amplified using primers S-D-Bact-0341-b-S-17 and S-D-Bact-0785-a-A-21 (Klindworth et al., 2013) with the addition of the Illumina overhang adapter sequences.

Before amplification, DNA extracts were normalized by means of Quantitative real-time PCR (qPCR) Ct values with the same amplification primer pairs and the same protocols described by Bruno et al. (2016, 2017). Library sequencing was performed through Illumina MiSeq instrument using MiSeq Reagent Kit v3 (2 × 300-bp paired-end sequencing). The library preparation and the sequencing process were conducted by the Center for Translational Genomics and Bioinformatics of Hospital San Raffaele (Milan, Italy).

### Microbial Composition and Community Structure Analysis

Analysis of bacterial communities was performed using the plugins of the QIIME2 suite (Caporaso et al., 2010). Raw Illumina reads were paired and pre-processed using VSEARCH v2.5.0 *–merge pairs* algorithm (Rognes et al., 2016). Reads were filtered out if ambiguous bases were detected and lengths were outside the bounds of 250 bp. Moreover, an expected error = 1 was used as an indicator of read accuracy.

Bacterial features were obtained using the *–cluster_fast* algorithm with a 97% sequence identity (Rognes et al., 2016). In order to reduce the possible biases introduced during amplification and sequencing phases, we decided to consider only those clusters having more than 15 reads in the dereplication step and more than five sequences in the *de novo* clustering step. In both cases, we used the VSEARCH software to conduct the analysis. The cluster centroid for each feature was chosen as the representative sequence of the cluster. The taxonomic assignment of the representative sequences, to obtain the Operational Taxonomic Units (OTUs), was carried out using the *feature-classifier*^1^ plugin implemented in QIIME2 against the SILVA SSU non-redundant database (128 release) adopting a consensus confidence threshold of 0.8.

The intra group diversity (alpha diversity) was calculated using the number of observed OTUs and the Faith’s Phylogenetic Diversity (Faith, 1992), evenly sampled at 1,000 reads per sample. The Kruskal–Wallis (pairwise) test was used to test for associations between discrete metadata categories and alpha diversity data.

Community analyses (beta diversity) were performed with qualitative (Jaccard and unweighted UniFrac; Lozupone and Knight, 2005) and quantitative (Bray-Curtis and weighted UniFrac; Lozupone et al., 2007) distance metrics (evenly sampled at 1,000 reads per sample) using the *diversity* QIIME2 plugin. Statistical significance among groups (sampling site and cultivar) was determined by the ADONIS (permutation-based ANOVA, PerMANOVA) test (Anderson, 2005) with 999 permutation-based Bray-Curtis distance matrices. PerMANOVA Pairwise contrast was performed by the beta-group-significance command of *diversity* plugin. We decided to adopt an ordination approach to explore the structure of microbial communities and specifically, we used principal coordinates plots (PCoA). The phylogenetic tree necessary to calculate UniFrac distances and Faith’s Phylogenetic Diversity was based on the alignment of OTUs representative sequences. The tree was built using RAxML version 7.4.2 (Stamatakis, 2006) with the GTRGAMMA model bootstrapping (1,000 replicates) the best maximum likelihood tree inference. Multibar plots were generated with the QIIME2 dedicated plugin *taxa*^2^.

The Venn diagrams were created with the online tool^3^. This tool allows to calculate the intersection(s) of list of elements that in this study was represented by the list of genera of bacteria found in each sample (i.e., soil and grape cultivars) at each site. The tool generates a textual output indicating which elements are in each intersection or are unique to a certain list and produces a graphical output in the form of a Venn/Euler diagram.

The Random Forest classifier implemented in the *sample-classifier* QIIME2 plugin^4^ was used to predict a categorical sample metadata category (i.e., sampling site, cultivar, and the combination of the two variables). The number of trees to grow for estimation was set to 1,000. Overall accuracy (i.e., the fraction of times that the tested samples are assigned the correct class), was calculated for each factor. K-fold cross-validation was performed during automatic feature selection and parameter optimization steps. A fivefold cross-validation was also performed.

To evaluate which components of grape and soil microbiome mostly contribute to the correct prediction of cultivar-provenance cases, we generated a heat map representation of the significant discriminatory features (bacteria genera) selected by the machine learning analysis. Samples and features axes where also organized by using a clustering approach. The heat map was generated with the *feature-table* QIIME2 plugin (McDonald et al., 2012).

## Results

### Sequence Analysis

Samples of grape and soil were sequenced in replicate. After filtering and primer removal, the remaining sequences were of high quality and had an average length of 430 bp (range: 400–438 bp) and clustered into 1154 OTUs (Supplementary Data S1). To characterize the microbial consortia associated with grapes of the three cultivars (45 samples) and soil samples (15 in total), 2,056,066 and 1,450,304 quality-filtered 16S rRNA sequences were obtained, respectively. After the removal of sequences corresponding to the grapevine genome (mitochondrial and chloroplast genomes included), and singleton sequences, a total of 818,076 and 1,001,230 sequences were used to describe the microbial profile of grape and soil samples.

### Grape and Soil Microbiome Diversity and Distribution

The microbial taxonomic composition of grape samples encompasses a total of 18 phyla (Bacteria domains), 55 classes, 98 orders, 197 families, and 374 genera. Soil samples revealed a complex microbiome with a total of 22 phyla (Archaea and Bacteria domains), 64 classes, 111 orders, 203 families, and 365 genera.

Regardless of provenance and cultivar, grape bacterial communities were dominated by Proteobacteria (71.4%), Firmicutes (12.7%), Actinobacteria (9.6%), Bacteroidetes (3.4%). Complete taxonomic assignments for each detected OTU are shown in Supplementary Data S1.

The most abundant classes of bacteria, Alphaproteobacteria, Betaproteobacteria, Gammaproteobacteria, Clostridia, Bacilli, and Actinobacteria, were shared by all cultivars and sampling localities. Conversely, other less abundant classes, Cytophagia, Sphingobacteria, Cyanobacteria, Acidimicrobiia, Blastocatellia, Thermoleophilia, Erysipelotrichia, Deltaproteobacteria, and Flavobacteria, were shared mainly by grapes from Italian Localities NI and AI (Supplementary Data S1).

To better explore the microbial differences among localities, grape cultivars and soil, we computed beta diversity metrics and generated PCoA plots. In order to normalize the variance during the analysis, we set the even sampling depth to 1,000. The script that calculates beta diversity metrics uses this parameter to subsample the counts in each sample without replacement, so each sample in the resulting table has a total count of 1,000. If the total count for any sample is smaller than 1,000, the samples are dropped for the diversity analysis. Using this value, we lost two SB and three SY samples from NS.

**FIGURE 1:**
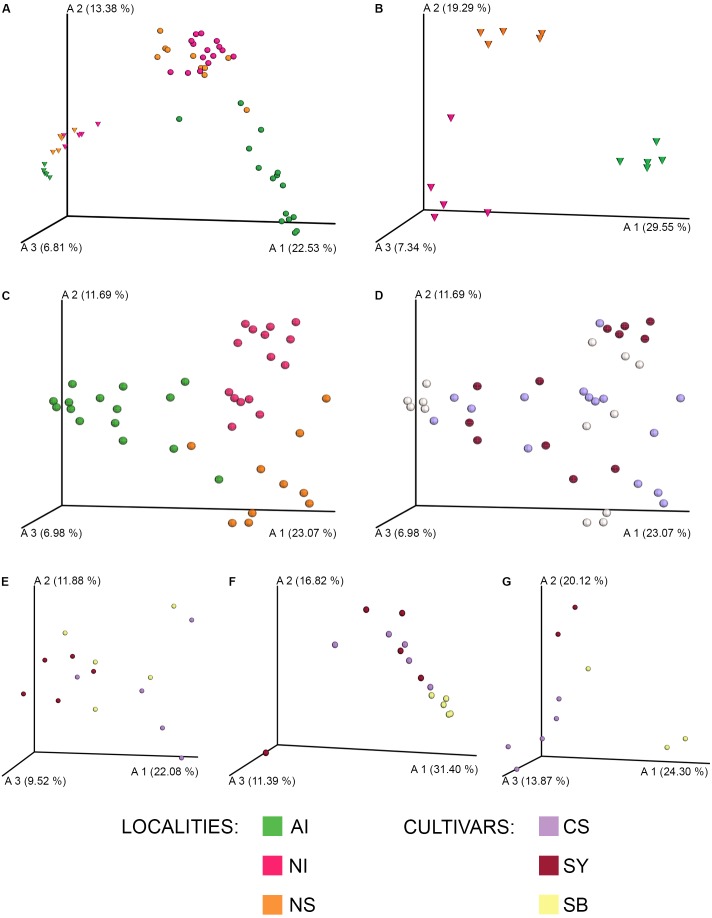
PCoA Emperor plots based on Bray–Curtis diversity metric. For each sampling locality **(A–C)** and cultivar **(E–G)**, samples of soil (triangles) and grape (circles) were compared concerning their microbial community. **(A)** Overall comparison of grape and soil samples; **(B)** soil samples; **(C)** grape samples; **(D)** overall comparison among grape cultivars; **(E)** cultivars from the Lombardy regional collection (Northern Italy, NI); **(F)** cultivars from the germplasm collection of E. Mach Foundation (AI, Alpine Italy); **(G)** cultivars from the La Rioja collection (NS, Northern Spain). CS, Cabernet Sauvignon; SY, Syrah; SB, Sauvignon Blanc.

The overall PCoA (**Figure 1A**) depicts a neat separation between soil and grape microbiomes. This pattern is not due to the high similarity within grape or soil samples, because when we stratified for both categories (**Figures 1B,C**) a significant geographical structuration occurred (for soil: NI *vs* AI, *pseudo-F* = 5.80, *p* < 0.001; NI *vs* NS, *pseudo-F* = 3.48, *p* < 0.001; AI *vs* NS, *pseudo-F* = 5.92, *p* < 0.001; for grape: NI *vs* AI, *pseudo-F* = 9.73, *p* < 0.001; NI *vs* NS, *pseudo-F* = 4.46, *p* < 0.001; AI *vs* NS, *pseudo-F* = 7.91, *p* < 0.001).

No significant correlation was detected between the cultivars and their microbiome profile (p > 0.05) considering the overall sampling panel (**Figure 1D**, Supplementary Data S2 for complete PERMANOVA pairwise test results). However, a slight structuration of cultivars was detected at each locality. In the case of NI, only CS and SY were significantly different (*pseudo-F* = 2.21, *p* < 0.01), whereas no significant differences occurred for SB *vs* SY and CS *vs* SB (p > 0.05) (**Figure 1E**). In AI, the SB samples were significantly different from the red-grape cultivars (SB *vs* CS, *pseudo-F* = 3.12, *p* < 0.001; SB *vs* SY, *pseudo-F* = 3.97, *p* < 0.01), while no significant differences were observed for CS *vs* SY (*p >* 0.05) (**Figure 1F**). Similarly, Spanish grape samples (NS) showed a significant difference between the microbiome profile of SB and CS (*pseudo-F* = 2.22, *p* < 0.01), while the other comparisons did not show supported differences (i.e., SB *vs* SY and CS *vs* SY, *p* > 0.05) (**Figure 1G**).

### The Origin of the Grape Microbiome

**FIGURE 2:**
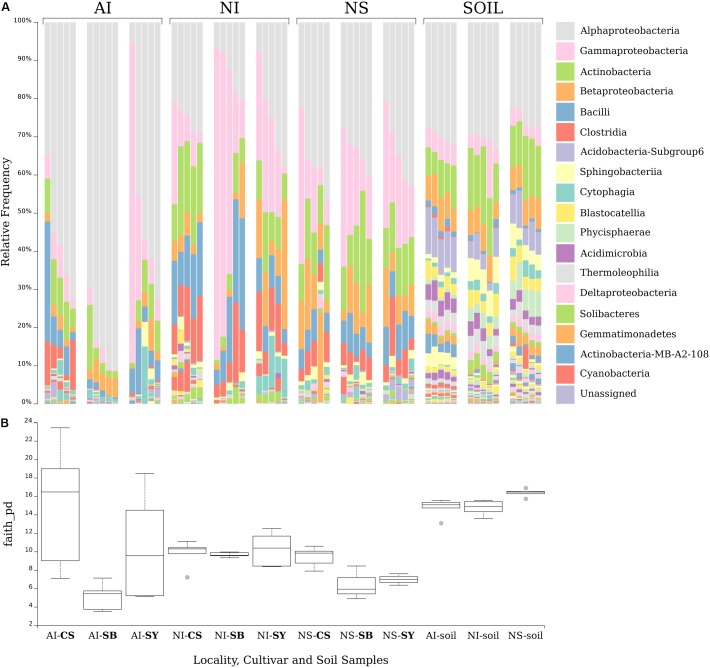
Bar chart analysis depicting the relative abundance and distribution of the OTUs assigned to class taxonomic rank **(A)**. The legend lists the 18 most abundant Classes. Boxplots **(B)** show the Faith’s Phylogenetic Diversity (a qualitative measure of community richness incorporating phylogenetic relationships) for each cultivar’s grape and soil sample at each sampling site. Localities and cultivars are reported with the same acronyms detailed in the manuscript and in **Figure 1** caption.

**Figure 2A** shows the distribution of bacterial classes per grape having a relative abundance >0.005%. Cultivar, geographical provenance variables, and microbiome data from soil samples are included as well. Compared to grape, soil was richer in terms of microbial diversity (**Figure 2B**; PD metric (mean +/- sd): grape = 9.56 +/- 4.13; soil = 15.3 +/- 1.05; H = 20.4; *p* < 0.0001). Several of the most abundant bacterial classes were shared between soil and grape samples (i.e., Alphaproteobacteria, Gammaprotecobacteria, Actinobateria, and Betaproteobacteria) (**Figure 2A**). However, microorganisms belonging to Bacilli and Clostridia (Firmicutes) occurred more frequently on grape surface than in soil samples.

Concerning the microbial diversity among localities, the alpha diversity analysis suggested that, in general, Spain grape samples (NS) had the lowest microbial diversity [PD metric, AI: (mean +/- sd) = 18.99 +/- 9.14; NI: (mean +/- sd) = 13.71 +/- 3.39; NS: (mean +/- sd) = 6.71+/- 4.55; H = 2.76; *p* = 0.009]. **Figure 2B** showed that some samples of NS (SB and SY) had a microbial diversity lower than others (**Figure 2**, pairwise Krustal–Wallis test results are reported in Supplementary Data S3).

Moreover, the abundance of some bacterial classes such as Clostridia (Firmicutes) was lower in NS samples of Sauvignon Blanc and Syrah.

To assess which bacterial genera were exclusive for a certain cultivar and/or locality and to evaluate the influence of soil bacteria in modeling grape microbiome, we estimated the portion of shared genera between soil samples and related grape cultivars at each sampling site.

**FIGURE 3:**
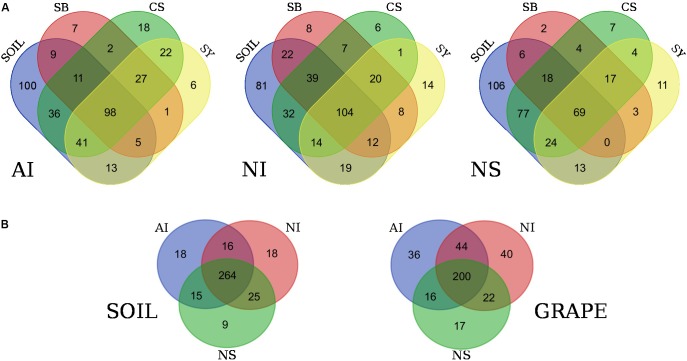
Venn diagrams showing the number of shared bacterial genera among grape cultivars and soil at each locality **(A)**. In **(B)**, the diagrams show the number of shared bacterial genera among soil samples and grape samples (regardless of cultivar) from the three localities. Localities and cultivars are reported with the same acronyms detailed in the manuscript and in **Figure 1** caption.

Venn diagrams confirmed that each cultivar shared almost 60% of genera with soil (**Figure 3A**). Specifically, in the case of AI the number of soil genera shared with cultivars ranged from 186 in CS to 123 in SB. At NI, they ranged from 189 in CS to 139 in SY and at NS, and 188 and 93 genera were shared between soil and CS and SB respectively. Interestingly, some unique microbial traits were found. Most bacterial genera were shared by all cultivars but were exclusive to a certain sampling locality; however, other genera were unique to single cultivars. For example, in the case of AI, 27 genera were shared among the three cultivars but not with the soil microbiome, while 7, 18, and 6 were unique to SB, CS, and SY, respectively. A similar situation occurred for NI vineyards where 20 bacterial genera were shared among the three cultivars while 8, 6, and 14 were unique to SB, CS, and SY, respectively. Concerning NS, 17 genera were shared among the three cultivars while 2, 7, and 11 were unique to SB, CS, and SY, respectively (**Figure 3A**).

**Figure 3B** shows the number of shared bacteria genera among all soil samples and among grape samples (regardless of cultivar). Overall, data show that soils of the three localities share a greater proportion of bacterial genera than those shared among cultivars at the same site. At the taxonomic level, grape samples of the three localities show 36, 40, and 17 genera exclusive to AI, NI, and NS, respectively. The complete list of these particular bacteria is reported in Supplementary Data S4.

### Machine Learning Analysis

**FIGURE 4:**
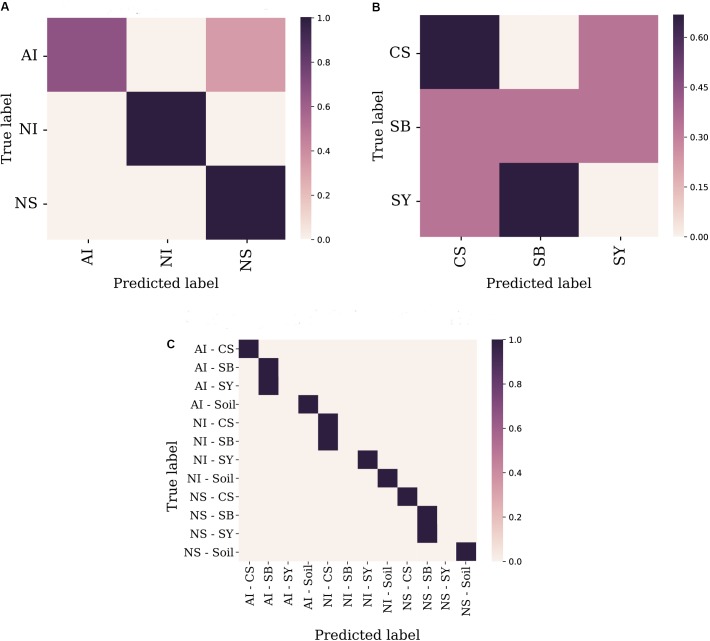
Machine learning analysis performed at the sampling locality level **(A)**, cultivar identity **(B),** and the combination of the two factors **(C)**. Features’ table was collapsed to the genus level. Overall accuracy levels are indicated as a scatter plot showing predicted vs. true values for each tested sample, along with a linear regression line fitted to the data with 95% intervals (gray shading). Localities and cultivars are reported with the same acronyms detailed in the manuscript and in **Figure 1** caption.

A random forest was used as supervised learning classifier to predict cultivar identity and provenance of a certain grape sample based on its microbiome composition. Taxonomic diversity at the genus level (or at the most informative taxonomic rank when the genus was not available), was used as a trainer for the classifier. At the geographical level, the comparison between “true label” *vs* “predicted label” showed the highest probability to correctly predict the geographical origin of NS and NI grape samples, while the overall accuracy reached a value of 0.75 in the case of AI accessions (**Figure 4A**). Conversely, the prediction level for cultivar identity showed higher uncertainty with the only exception of CS which reached about 0.6 of overall accuracy (**Figure 4B**). When combining the two factors (i.e., sampling site and cultivar identity), the machine learning tool correctly predicted 9 cases out of 12 with high accuracy (**Figure 4C**). Accuracy values for each of the three tested models are reported in Supplementary Data S5.

**FIGURE 5:**
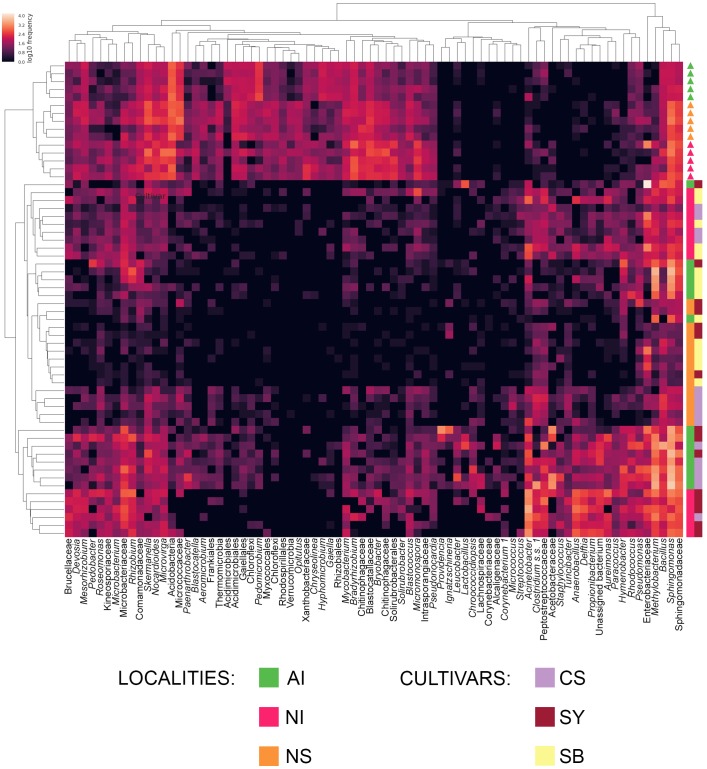
Heat map showing the relative abundance of the components of grape and soil microbiome mostly contributing to the correct prediction of cultivar-provenance cases in the machine learning analysis. If available, the genus level was considered (italic). In the other cases, we reported the most informative and supported taxonomic rank returned by the *classify-consensus-vsearch* plugin. Color shading in the heat map indicates the abundance (expressed as log10 frequency) of each genus in the sample. The upper cladogram, shows groups of bacteria genera based on their distribution among samples, whereas the left cladogram shows clusters of grape samples based on genera distribution. The provenance and cultivar of each soil (triangles) and grape (squares) sample are reported on the right of the heat map. Localities and cultivars are indicated with the same acronyms and colors detailed in the manuscript and in **Figure 1** caption.

To evaluate which components of grape and soil microbiome mostly contributed to distinguish the nine correctly predicted cultivar-provenance cases, we used a heat map diagram (**Figure 5**). This analysis indicates that at least 87 bacterial genera act as significant features explaining the results of the combined random forest model of **Figure 4C**. Specifically, some combinations of locality-cultivar, such as NI_SY, AI_CS, and NS_CS, formed well-defined clusters. The indecision case involving AI_SB and AI_SY (**Figure 4C**) is not supported by the heat map which shows that AI_SB samples cluster together with only one AI_SY sample (**Figure 5**). This means that the missing prediction of AI_SY samples in the machine learning analysis is due to the high and heterogeneous microbial diversity of SY individuals.

Conversely, the heat map shows that NI_CS - NI_SB and NS_SB - NS_SY samples are scattered in two different clusters, thus supporting the failed prediction emerging from the random forest analysis.

## Discussion

### Vineyards as a Complex and Dynamic Ecosystem

This study supports the hypothesis that vineyard soil represents a primary reservoir for grape associated bacteria, most of which are involved in processes ranging from plant nutrition and development to the modification of grape and wine quality (Burns et al., 2015; Bokulich et al., 2016; Belda et al., 2017). For example, in our samples, several members of Alphaproteobacteria (e.g., Rhizobiales, Rhodobacterales, Sphingomonadales) were shared between the soil and grape at each investigated locality. Rhizobiales (e.g., *Bradyrhizobium*) contribute to plant nutrition, since they are involved in nitrogen fixation. Although in many cases these bacteria form root nodules, some species may be found in other plant portions and could provide nutrients to the plant even though it lacks nodules (Bacon and White, 2016). Among Rhodobacterales and Betaproteobacteria, we detected members of *Craurococcus* (Acetobacteraceae) and *Massilia* that are involved in the metabolism of phosphate and in plant growth promotion, respectively (Ofek et al., 2012; Kecskeméti et al., 2016). Other important microbial taxa found on the grape surface and belonging to soil were Bacillales and Clostridiales (Zarraonaindia et al., 2015). In some cases, the occurrence of these bacteria is related to the fertilization strategy, including the use of manure (Ding et al., 2014). Their role in fruit is not clear yet; however, it is known that these microorganisms also persist during vinification, thus it is expected that they can influence fermentation processes and wine quality (Piao et al., 2015). Furthermore, soil bacteria such as *Gluconobacter* (Alphaproteobacteria) were also found in our grape samples at all the investigated localities. These are expected to play an important role during wine fermentation depending on the developmental phase of grape at the moment of harvest and could affect wine quality as well.

Given the pivotal and renowned importance of the soil microbiome in the era of precision agriculture, any tool able to enhance the occurrence of key microorganisms on grape surfaces could really have an impact on wine quality (Burns et al., 2016). For example, Martins et al. (2013) suggested that soil bacteria could easily reach the grape surface during rain or when transported by wind. Therefore, the currently adopted precision irrigation systems could enhance or reduce soil microorganism colonization rate (Campos et al., 2000) and favor the movement of bacteria from soil to the fruit. Other practices, such as the use of cover crops could also influence soil microbial ecology and indirectly grape microbiome (Ingels et al., 2005).

The findings discussed here provide new information concerning the microbial diversity of vineyard soils. Microbial diversity was high in our samples, but there were a few differences among the three investigated sites. Particularly, differences were due to bacteria involved in processes such as degradation organic matter (e.g., *Azoarcus*) and fertilization (e.g., *Larkinella*). Moreover, parasitic bacteria (e.g., *Burkholderia* and *Serratia*) also occurred. This condition agrees with the idea that vineyard soils could share a core of bacteria but differ in those microbial groups more influenced by the biotic/abiotic factors of the vineyard, including farming management (Pinto and Gomes, 2016). In general, these “extra-core” bacteria seem to not influence the grape microbiome. For example, among the 36 microbial genera unique to the site in AI grape samples, only three (*Luteibacter*, *Spirosoma*, and *Taibaiella*) were shared with the soil. In the cases of NI and NS samples, we did not find any shared microbial genus between soil and grape within those bacterial genera unique to each site. Conversely, the microbial core of soil could have a greater influence on the bacterial genera on grape at each sampling site, as we found from 41% to 88% genera of AI and NI grape, respectively, to belong to soil-core OTUs. The remaining genera could have an extra-soil origin. Some of these bacteria (e.g., *Wolbachia*, *Cardinium*, *Rickettsia*, and *Hamiltonella*) could belong to arthropods in vineyards (Delort and Amato, 2017), thus supporting the hypothesis of a functional role played by local biodiversity in transferring microbial organisms to the grape (Gilbert et al., 2014). A similar situation has been demonstrated for grape yeasts (and their strains) which were found to disperse and evolve using wasps and other hymenopterans as vectors (Stefanini et al., 2012; Dapporto et al., 2016).

Vineyard structure and management could indirectly act on the process of microbial transfer by influencing the communities of potential vectors inhabiting this agroecosystem, such as insects (Sanguankeo and León, 2011; Caprio et al., 2015) and birds (Assandri et al., 2017a,b), at a multilevel scale. These animals use the vineyard as part of their home range as trophic or reproductive niche, also favoring the introduction of microbes from other habitats.

### The Role of the Grapevine and Vineyard Ecosystem in the Selection of the Fruit Microbiome

One of the aims of this study was to evaluate the role of the grapevine in selecting the epiphytic microbial community of grape berries. We hypothesized that when microorganisms reach the berry, they establish and start to interact with fruit skin. These dynamics occur between the external waxy layer (bloom) of the berry, which is useful for preventing water loss through evaporation, and the hypodermis layer (Knoche and Lang, 2017). It is known that the number of skin layers of grape berries and their thickness are cultivar-specific. Although in our case, the thickness of the three selected cultivars was similar, the natural waxy coat of CS is more abundant in comparison to SB, while SY shows an intermediate value (International Organisation of Vine and Wine, 2015). These physical features could influence the contact and permeability of the grape berry cuticle to different microorganisms as observed for some pathogens, such as *Botrytis cinerea* (Herzog et al., 2015). Moreover, also the occurrence of anthocyanins could have a role in shaping the grape microbiome due to the antimicrobial properties of this group of molecules (Cisowska et al., 2011; Apolinar-Valiente et al., 2017). In this study, anthocyanins occur only in the two dark berry cultivars in CS and SY (**Table 1**). Concerning bunch features, the three cultivars showed different densities, sizes, and shapes (**Table 1**); therefore, we expected that these traits could also have an influence on the access and permeability of microorganisms to the bunch. The PCoA and the machine learning analysis suggested that the pedoclimatic characteristics of sampling sites play a major role in selecting the microorganisms on grape surfaces rather than the plant ampelometric characteristics. Probably, local environmental conditions combined with agronomic management characteristics are more able to modify the berries microbiome, at least much more than the genetic characteristics of plants. This could explain why all grape cultivars at each locality shared a different fraction of soil-core bacteria.

However, our analyses (e.g., **Figure 5**) suggested that most SB accessions (especially from AI and NS) have some different microbial features if compared to the dark berry cultivars. For example, these latter share more bacterial genera with soil (e.g., *Devosia*, *Mesorhizobium*, *Rhizobium*, and *Pedobacter*) than the SB. Moreover, this white-grape cultivar does not share some common bacteria belonging to the genera *Anaerobacillus*, *Delftia* and *Propionibacterium* found on CS and SY samples. Microbiome’s differences between red- and white-grape cultivars were already showed by Bokulich et al. (2014) analyzing grape must samples of Cabernet Sauvignon and Chardonnay.

Given that, it is reasonable to assume that some plant and/or grape traits could serve as selecting agents of specific bacteria. This hypothesis also explains the higher (up to 75%) predictive power of machine learning analysis when considering a combination of factors (i.e., site and cultivar) rather than the single ones separately (**Figure 4C**).

## Conclusion

In the past, grapevine management and wine production exploited the experience and knowledge of wine growers and enologists who worked to optimize production based on agronomic and chemical parameters. Although the general principles of fermentation were known, wine organoleptic properties were usually attributed to the geographical origin of grape. In the last few years, the development and higher affordability of High Throughput Sequencing (HTS) technologies allowed a better understanding about the microbial dynamics involving the grapevine, from the field to the barrel. By taking advantage of HTS technologies in this study, we suggest a key role of soil and vineyard biodiversity in influencing the grape microbiome and a secondary but heterogeneous role of the grapevine. Although this kind of research could provide valuable information on wine origin, the interpretation of HTS microbiome data deserves caution, because there are still unknown interactions between plants and environmental microorganisms. Further difficulties reside in the possibility of recovering a large amount of data that is representative of seasonal and geographical changes. It should also be highlighted that the analytical potential of molecular tools and the standardization of bioinformatics pipelines combined with the emerging machine learning approaches offer new opportunities to develop wider and integrated research activities to test which variables have an effective role in shaping microbiome composition and dynamics over time and space. These perspectives will also permit an efficient integration with metabolome features of grapevine accessions to uncover the intimate sensorial characteristics of grapes and wine.

## Data Availability

The datasets generated for this study can be found in the EBI metagenomics portal (https://www.ebi.ac.uk/metagenomics/) under the accession code PRJEB25720 (ERP107664).

## Author Contributions

VM, AS, and ML conceived and designed the experiments. VM performed the experiments. AS, LG, and ML analyzed the data. MG and JT contributed materials. VM, AS, AG, and ML drafted the manuscript and figures. All authors contributed to the revision of the final manuscript.

## Conflict of Interest Statement

The authors declare that the research was conducted in the absence of any commercial or financial relationships that could be construed as a potential conflict of interest.

## Footnotes

1 https://github.com/qiime2/q2-feature-classifier

2 https://github.com/qiime2/q2-taxa

3 http://bioinformatics.psb.ugent.be/webtools/Venn/

4 https://github.com/qiime2/q2-sample-classifier
