# Supplementary material for: Geographical and Cultivar Features Differentiate Grape Microbiota in Northern Italy and Spain Vineyards
Source: Front Microbiol. 2018 May 15;9:946. doi: 10.3389/fmicb.2018.00946 (PMC5962658; doi:10.3389/fmicb.2018.00946)
Supplement: Supplementary file 1 [file Data_Sheet_1.ZIP › Supplementary_Material_README.docx]

***Supplementary Material***

Geographical and cultivar features differentiate grape microbiota in Northern Italy and Spain vineyards

**Valerio Mezzasalma^☨^, Anna Sandionigi^☨^, Lorenzo Guzzetti, Andrea Galimberti, Maria Stella Grando, Javier Tardaguila, Massimo Labra***

^☨^ These authors contributed equally to this work.

###### * Correspondence:

Dr. Massimo Labra

massimo.labra@unimib.it

# Supplementary Data

**Supplementary_Data_S1.csv**

OTUs Assignment. For each unique OTU, (Feature ID), assigned Taxonomy and the confidence value of the assignment are reported.

**Supplementary_Data_S2.csv**

PERMANOVA pairwise results considering as response variable Bray-Curtis dissimilarity matrix.

**Supplementary_Data_S3.csv**

Krustal-Wallis pairwise test with measure of Faith PD metric as response variable. Samples are clustered for Cultivar and Geographic Origin.

**Supplementary_Data_S4.csv**

Multi-sheet file including all the Venn diagram results. The results of each diagram depicted in Figure 4 are reported in a separate sheet.

**Supplementary_Data_S5.csv**

Machine learning overall accuracy. Classification accuracy results for the three tested models showed in Figure 5.

**Supplementary_Data_S6.txt**

Significant discriminatory features (i.e., bacteria genera) selected by the machine learning analysis.
